# Dosimetric precision of an ion beam tracking system

**DOI:** 10.1186/1748-717X-5-61

**Published:** 2010-06-30

**Authors:** Christoph Bert, Alexander Gemmel, Nami Saito, Naved Chaudhri, Dieter Schardt, Marco Durante, Gerhard Kraft, Eike Rietzel

**Affiliations:** 1 GSI Helmholtzzentrum für Schwerionenforschung GmbH, Abteilung Biophysik, Planckstraße 1, 64291 Darmstadt, Germany; 2 Siemens AG, Healthcare Sector, Imaging & Therapy, Particle Therapy, Hofmannstr. 26, 91052 Erlangen, Germany

## Abstract

**Background:**

Scanned ion beam therapy of intra-fractionally moving tumors requires motion mitigation. GSI proposed beam tracking and performed several experimental studies to analyse the dosimetric precision of the system for scanned carbon beams.

**Methods:**

A beam tracking system has been developed and integrated in the scanned carbon ion beam therapy unit at GSI. The system adapts pencil beam positions and beam energy according to target motion.

Motion compensation performance of the beam tracking system was assessed by measurements with radiographic films, a range telescope, a 3D array of 24 ionization chambers, and cell samples for biological dosimetry. Measurements were performed for stationary detectors and moving detectors using the beam tracking system.

**Results:**

All detector systems showed comparable data for a moving setup when using beam tracking and the corresponding stationary setup. Within the target volume the mean relative differences of ionization chamber measurements were 0.3% (1.5% standard deviation, 3.7% maximum). Film responses demonstrated preserved lateral dose gradients. Measurements with the range telescope showed agreement of Bragg peak depth under motion induced range variations. Cell survival experiments showed a mean relative difference of -5% (-3%) between measurements and calculations within the target volume for beam tracking (stationary) measurements.

**Conclusions:**

The beam tracking system has been successfully integrated. Full functionality has been validated dosimetrically in experiments with several detector types including biological cell systems.

## Background

At GSI Helmholtzzentrum für Schwerionenforschung (GSI) more than 430 patients with tumors mainly in the head and neck area were treated with a rasterscanned carbon beam [1,2]. For treatment of respiration-influenced tumors motion mitigation techniques will be required because the interference of target motion and scanned beam delivery potentially leads to mis-dosage, typically referred to as interplay [3,4]. Beam gating [5], rescanning [3], and beam tracking [6,7] have been proposed to adequately irradiate moving targets with scanned particle beams.

Tracking has been suggested in different technical ways and for different treatment modalities. For photon radiotherapy tracking is implemented clinically in the Cyberknife Synchrony system [8]. Adaptations are primarily in the lateral dimensions and can therefore also be performed by dynamically adapting the multi-leaf collimator of a standard linear accelerator [6]. In contrast to photon therapy, particle therapy requires modulation not only in the lateral direction but also in the radiological depth because organ motion potentially changes densities in the beam paths and therefore the particle ranges [9].

A feasibility study at GSI showed that the rasterscan beam delivery system can be extended to treat moving tumours by beam tracking by adapting the position of rasterpoints [10]. Lateral adaptation is performed by real-time changes of the scanning magnet settings. Compensation of changes in radiological depth is carried out by a passive energy modulation system installed proximal to the isocenter. The system consists of two opposing absorber wedges that are opened (closed) by fast linear motors when the radiological length has to be increased (decreased). Within the feasibility study, individual compensation components were tested independently. To allow simultaneous lateral and range adaptation the initial prototype system has been redesigned, fully integrated into the therapy control system (TCS), and technically commissioned [7,11].

The data in this report present a full set of dosimetric studies performed with the most recent version of the tracking system. Earlier investigations focused on individual components of the beam tracking system [10], its technical performance [11], as well as initial dosimetric measurements [7]. We utilized our experience from previous, independent measurement series to determine the accuracy of 3D dose distributions as well as the RBE-effective dose, to investigate the implications of beam tracking for volumes proximal to the target volume, and to perform detailed measurements with respect to range adaptation. In order to examine the beam tracking performance independent from possible ambiguities of target motion detection an accurate industrial motion sensor was employed to monitor the motion trajectories of moving phantoms.

## Methods

### Experimental setup

Four different detector types were used to test dose delivery by the integrated beam tracking system: radiographic films, a range telescope, an array of 24 ionization chambers, and biological cell samples. This combination was selected to measure the most important characteristics of particle dose distributions. (i) Radiographic film measurements provide high spatial resolution at a specific depth, (ii) the range telescope enables precision depth dose distribution measurements, (iii) the array of ionization chambers facilitates 3D measurements, and (iv) the biological cell samples allow judgment of the validity of the RBE-weighted dose.

**Figure 1:**
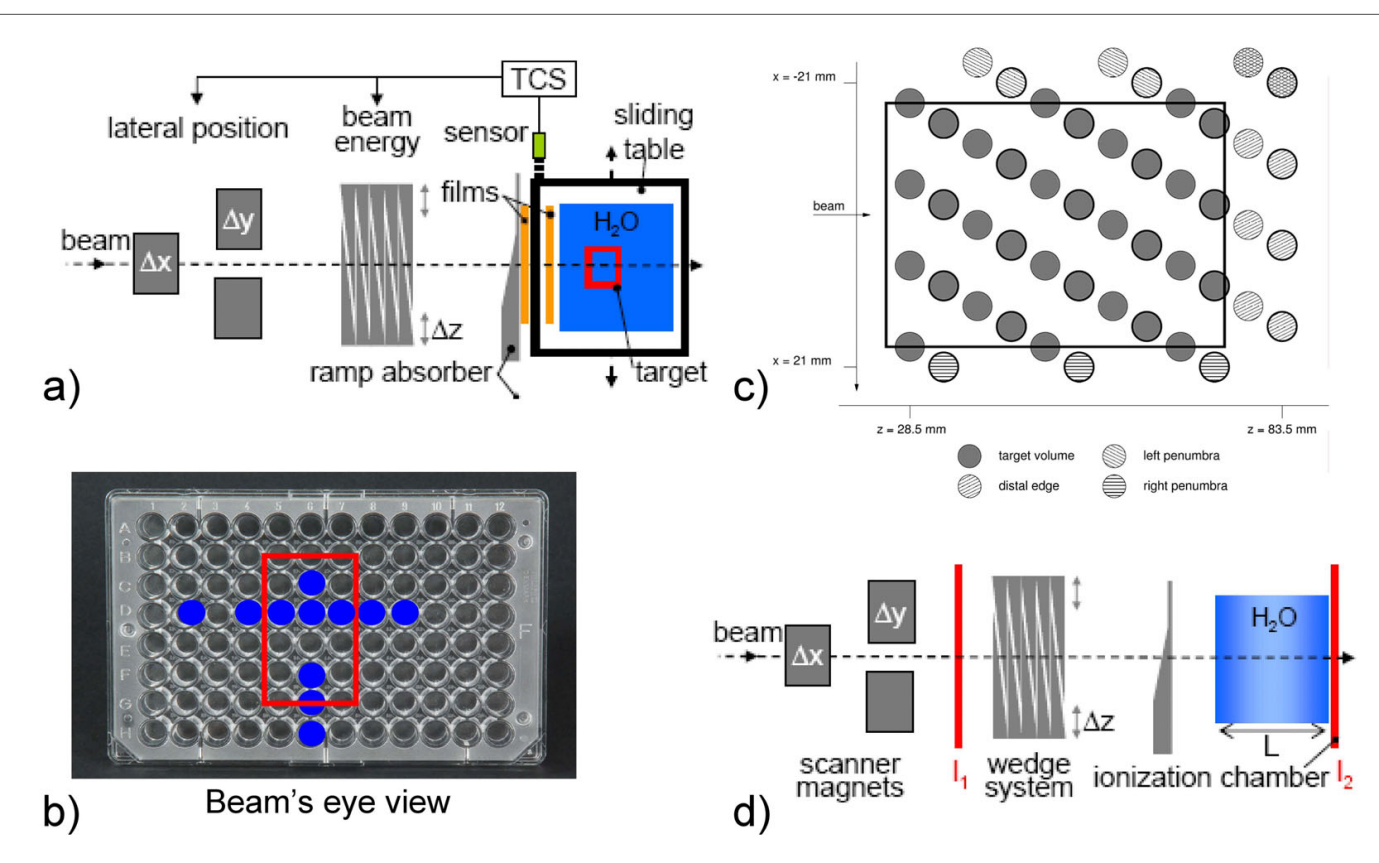
**Experimental setup**. Schematic drawing of the experimental setups. For film, cell sample, and ionization chamber experiments the target was moved on a sliding table left-right in beam's eye view (BEV). Proximal to the target, a ramp-shaped absorber was installed stationary such that lateral compensation induces range changes since the beam traverses this absorber at a different thickness. Films were positioned stationary directly behind the absorber as well as on the sliding table. The 24 ionization chambers are mounted within a water tank that is positioned on the sliding table. Data were acquired at two array positions as shown in (c) as bold and regular circles. For cell survival measurements two MicroWell plates where used with cell survival measurements performed at the positions indicated in (b). (d) For range validation, a range telescope in the target area was used to measure the relative ionization of two parallel-plate ionization chambers (I1 and I2). Range changes were induced as described in (a).

The experimental setup is shown in fig. 1. Besides the integrated beam tracking system, a sliding table was used to induce target motion. The motion was orthogonal to the beam direction, one-dimensional (left-right in beam's eye view), sinusoidal with an adjustable amplitude and period, and had a random starting point (motion phase) (details in tab. 1). Motion monitoring of the sliding table was performed with a laser triangulation displacement sensor [11]. In order to generate motion-induced variations in particle range, a stationary, ramp-shaped absorber was placed proximal of the sliding table (fig. 1a). If the particle beam position is adapted left-right to compensate lateral target motion the beam penetrates this ramp-shaped absorber at different positions with different thicknesses in comparison to the reference scenario. Compensation of these thickness changes had to be performed with the energy modulation system (see 4D treatment planning details by Bert & Rietzel [12]). In principle, this setup represents relative motion of different densities within a treatment field, for example lung tumors and ribs even though ribs might produce more discrete range changes.

**Table 1:** Treatment plan and delivery details for the different experiments.

**experiment**	**film response/cell samples**	**pin-point ionization chamber array**	**DDD**
	
target volume [WxHxD mm^3^]	28 × 45 × 23	36 × 20 × 50	single beam
depth range [MeV/u]	265 - 299	267 - 330	400
number of iso-energy slices	18	21	-
number of raster points	3444	5946	1
grid spacing [mm]	2.5	2	-
scan path	up-down	up-down	-
beam spot FWHM [mm]	7.5	5.6	2.3
peak-to-peak amplitude lateral/radiological depth [mm/mm water]	40/15.2	30/11.4	40/15.2
motion period [s]	~ 3	~ 3.4	~ 3
target dose	6 GyE (3.3 Gy at target center)	2 Gy	-
irradiation time [s]	~ 140	~ 150	-

The setup for the measurements with the 24 pin-point ionization chamber array (IC03, Wellhöfer, Schwarzenbruck, Germany), the radiographic films (Kodak X Omat V, Kodak GmbH, Stuttgart, Germany), as well as for the measurements with the cell samples is shown in fig. 1a. Film and cell sample detector were used in a single irradiation. One radiographic film was installed stationary distal to the ramp shaped absorber; the second, moving film was placed on the sliding table proximal to the container of the cell sample probe.

Chinese Ovary cells (CHO-K1) were used to measure cell inactivation based on the assay by Puck and Marcus [13] as described by Gemmel et al. [14]. The CHO-cells were seeded into MicroWell™ plates (Nunc, Roskilde, Denmark, 12 × 8 wells per plate, diameter per well: 7 mm, grid spacing: ~9 mm, 10000 cells per well). MicroWell™ plates were chosen because they provide adequate cell culture conditions and with respect to biological dosimetry they allow good sampling of data points in the lateral plane (see fig. 1b). Two MicroWell™ plates were stacked in upright position in a container filled with medium to achieve measurements at two different points in depth (±4.5 mm from the target center). Due to limited incubator space ~10 wells were analyzed per plate (marked in fig. 1b).

Data acquisition and setup of the 24 pin-point ionization chambers were performed as described by Karger et al. [15]. The chambers were arranged within a volume of ~60 × 70 × 15 mm^3 ^fixed on a polymethylmethacrylate (PMMA) block in three different heights to avoid dosimetric shadowing effects (see fig. 1c). The complete block can be positioned by mechanical stages within a water tank (MP3, PTW, Freiburg, Germany) that was placed on the sliding table. We measured at two different positions (see fig. 1c, with bold circles indicating the 24 chambers of one array position) to have a higher spatial resolution. This results in 48 data points of which 33 are positioned within the target volume that is indicated by the rectangle in fig. 1c.

A second setup (fig. 1d) was used for depth dose distribution (DDD) measurements with a range telescope [16] to assess the precision of range compensation. The range telescope determines the DDD by measuring the charge ratio of the distal (I_2_) and a proximal (I_1_) ionization chamber for different water thicknesses *L *(see fig. 1d and [17]). At each water thickness level and for three consecutive measurements the charge generated in the ionization chambers was accumulated for an accelerator pulse of 2.2 s duration (~1.5·10^7 ^particles). As described above, range changes were induced by deflecting the beam laterally over the stationary ramp absorber. Because lateral motion was continuous during the measurements the induced range changes were uncorrelated to the water thickness of the range telescope.

### Treatment plans and delivery

Reference treatment plans were optimized with our in-house treatment planning system (TReatment planning for Particles, TRiP) [12,18,19]. For each setup a different plan was used; plan details are listed in table 1. For all detectors, measurements were performed for (i) a stationary setup (reference), (ii) a moving setup without beam tracking (not for cell sample detector), and (iii) a moving setup with beam tracking. The experiment with the CHO-cell cultures was independently repeated three times. Each time a stationary setup and a moving setup with beam tracking was irradiated. In addition to the irradiated containers identical containers were prepared that served as controls for stationary and moving setup, i.e. went through the same procedures as the irradiated containers but were not exposed to irradiation. After the irradiation the cell survival in the wells marked in figure 1b was determined by trypsinizing, counting, and re-seeding (three times per well) the cells at an appropriate number. After an incubation time of 7 days the colonies were stained and counted for each well.

Beam tracking parameters were derived analytically. For a given peak-to-peak motion amplitude the minimum and maximum voltages of the displacement sensor were measured prior to the experiments and stored in the treatment control system. During beam delivery, the control system converted the voltage from the displacement sensor into lateral motion compensation parameters relative to these calibration measurements. Similarly, compensation parameters for the radiological depth were determined by multiplying the lateral compensation parameters with the slope of the ramp absorber (0.38 mm water-equivalent for 1 mm left-right motion). To overcome the response of range modulation which is ~25 ms for 5 mm water-equivalent (WE) range shift, linear motion prediction was used as reported by Saito et al. [11].

### Data analysis

Data analysis was performed relative to the stationary reference results for each measurement series.

#### Film response

Films were processed as reported by Spielberger et al. [20] and evaluated by

• the 2D distribution

• horizontal profiles which are sensitive for detection of positional deviations as well as fluctuations in film intensity

• analyzing the relative film response in a central region of interest (20 × 30 mm^2^) by mean, standard deviation, homogeneity index (defined as 1-standard deviation/mean) as well as minimum and maximum averaged over a 5 × 5 mm^2 ^area.

#### Depth dose profiles

Depth dose distributions were analyzed regarding the depth and height of the Bragg peak. Data points show the mean results of the three measurements per thickness level, error bars represent one standard deviation.

#### Pin-point ionization chambers (absorbed dose)

For the relative dose data of the ionization chamber array, mean, standard deviation, and maximum deviation of the relative and the absolute relative dose deviation are reported. Analysis was performed for all ionization chambers, the subset of chambers that was positioned within the target volume, for the left and right penumbra, as well as for chambers distal of the target volume. To visualize these four-dimensional data (coordinates are: BEV left-right, BEV up-down, BEV, relative dose) the relative dose to each spatial dimension is plotted, i.e. three two-dimensional graphs.

#### Biological cells samples (RBE-weighted dose)

The survival data of the three independent experiments were combined. For each well *j *mean survival  and the standard error of the mean survival  where *σ*_*j *_is the standard deviation of the three measured survival levels are reported. Data for irradiation modalities stationary and beam tracking are combined to mean survival  and  and mean standard errors  and , respectively. In agreement with the dose calculation steps in treatment planning we further convert the survival data into RBE-weighted doses *D *_RBE _by using the linear-quadratic model with literature data for *α *and *β*:  with *α *= 0.228 Gy^-1 ^und *β *= 0.02 Gy^-2 ^according to Weyrather et al. [21]. Also for the dose values mean  and  and standard error  and  are reported. For quantification of the beam tracking accuracy we did three comparisons:

C1) Experiment vs. TRiP (stationary): to benchmark the accuracy of biological dosimetry in standard conditions, comparing the stationary experimental result to the prescribed dose as determined by TRiP based on the local effect model (LEM III) [22].

C2) Experiment vs. TRiP (beam tracking): experimental results with beam tracking were compared to the prescribed dose.

C3) Beam tracking vs. stationary (experimental): experimental results with beam tracking were compared to experimental results with a stationary phantom. The mean standard error of this comparison is determined by .

Data will be presented graphically as  for each of the three comparisons. Wells in the target volume and outside of the target volume were also separately analyzed.

## Results

### Film response

**Figure 2:**
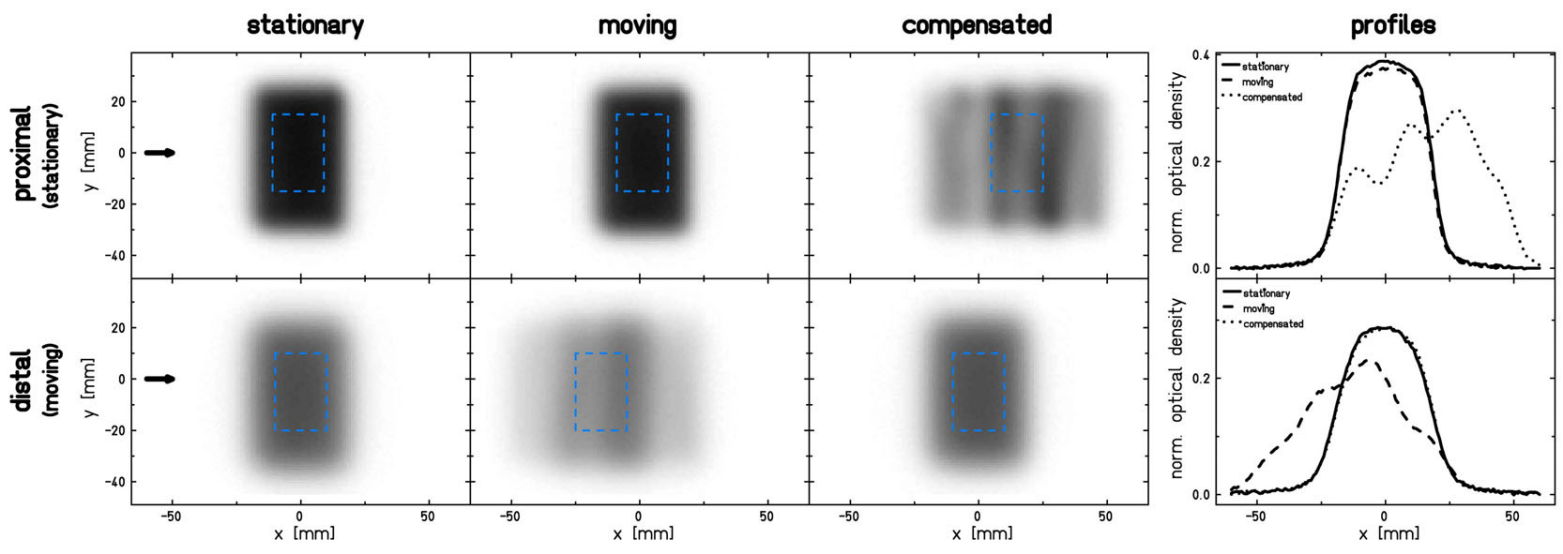
**Results of proximal and distal film responses**. Shown is the normalized optical density. Statistical analysis reported in table 2 was performed within the region of interest indicated by the dashed square. Horizontal profiles are in the direction indicated by the arrows (y = 0).

Results of the film response measurements are shown in fig. 2. For the stationary setup the irradiation results in a homogeneous response within the target area of both films. In case of target motion without beam tracking interplay distorts the film response distribution in the distal (moving) film. The response in the proximal (stationary) film is comparable to the response of the stationary measurement. With beam tracking, the results are vice versa: The result of the moving film is comparable to the stationary irradiation because beam adaptation compensates target motion. An "inverse interplay effect" caused by beam tracking of the moving target causes a deteriorated film response on the stationary film that resembles the path of the beam as it is adapted to the target motion. The horizontal profiles at the position indicated by the arrows confirm these results and indicate a slight shift to the right in BEV of the distal film for beam tracking in comparison to the stationary irradiation.

**Table 2:** Statistical analysis for the film response (normalized optical density).

		**proximal**			**distal**	
		
	**stat**.	**mov**.	**comp**.	**stat**.	**mov**.	**Comp**.
	
mean	0.38	0.37	0.26	0.28	0.20	0.28
SD	0.01	0.01	0.02	0.01	0.02	0.01
homogeneity	0.98	0.98	0.93	0.97	0.90	0.97
minimum_5 × 5_	0.36	0.34	0.21	0.24	0.17	0.25
maximum_5 × 5_	0.39	0.38	0.3	0.29	0.23	0.28

Statistical data in table 2 confirm that deviations between dose deliveries to a stationary target (mean 0.28, homogeneity 0.97) and to a moving target using beam tracking (mean 0.28, homogeneity 0.97) are comparable on the distal (moving) film.

### Depth dose profiles

**Figure 3:**
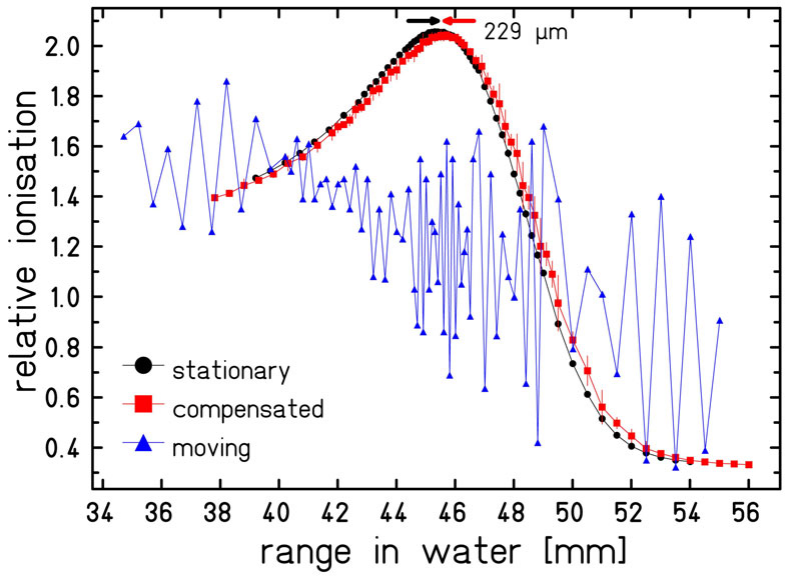
**Results of the range telescope measurements**. Measurements with a stationary setup, a moving setup without compensation, and a moving setup with compensation were performed. Mean and one standard deviation are plotted. The data points are connected to guide the eye.

Data of the DDD measurement are plotted in fig. 3. The influence of target motion on the shape of the Bragg curve is severe if no motion mitigation is applied. With beam tracking the DDD is comparable in shape and height to the stationary experiment. The peak depth is slightly (< 0.25 mm water-equivalent) shifted towards greater depth.

### Pin-point ionization chambers (absorbed dose)

**Figure 4:**
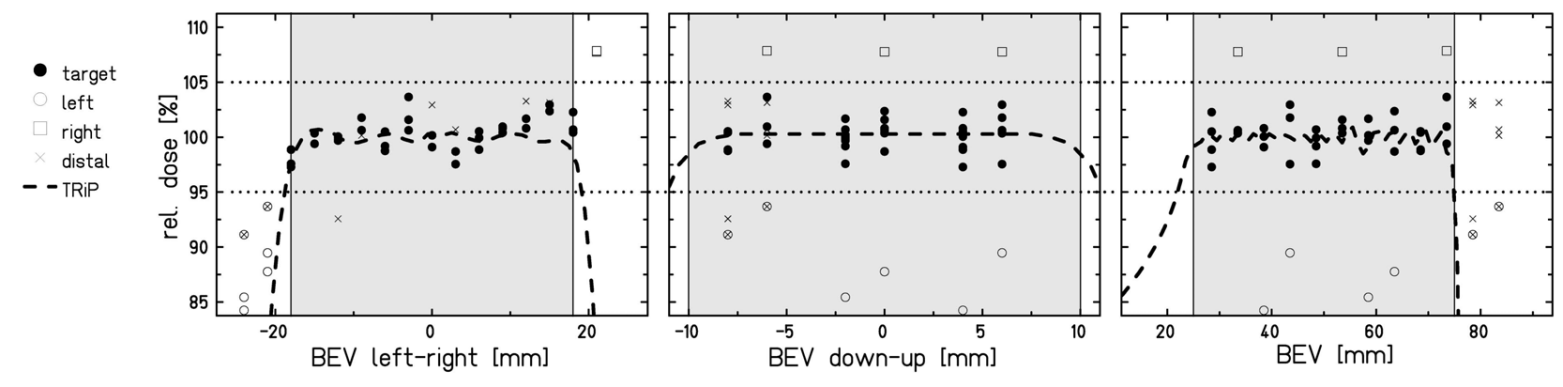
**Results of the pinpoint ionization chamber array measurements**. Shown are the projections of the beam tracking data measured by the ionization chambers. Data points are relative to the stationary reference irradiation. The dashed line indicates the nominal dose level; the two dotted horizontal lines indicate the 5% acceptance level, the shaded area indicates the target volume.

Results of the ionization chamber array measurements are displayed in fig. 4. With the exemption of three data points in BEV left-right (-24 mm, -21 mm, 21 mm), measured doses for tracking the moving target are within 5% in comparison to the stationary reference measurement. Within the target volume (33 of 48 chambers, corresponding to filled symbols in figures 1c and 4) doses delivered to a moving target with beam tracking deviated from the doses of a stationary reference irradiation by 0.3 ± 1.5% (abs. values: 1.2 ± 0.9%) with -2.7% minimum and 3.7% maximum deviation (details in table 3). A comparison of relative doses outside of the target volume indicates a small horizontal shift of measurement setups between reference and tracking experiment: left penumbra mean -13.3% and right penumbra mean + 7.8%. This shift occurred most likely due to a slight positional difference of the motion table between experiments translating into different ionization chamber positions. By minimizing the dose deviations at interpolated IC positions, a shift of -0.6 mm was determined.

**Table 3:** Statistical analysis for the ionization chamber array measurements.

IC position	Mean	SD	abs. mean	abs SD	minimum	maximum
target volume	0.3	1.5	1.2	0.9	-2.7	3.7

left penumbra	-13.3	2.3	13.3	2.3	-15.8	-10.5
right penumbra	7.8	0.1	7.8	0.1	7.8	7.9
distal edge	-1.5	5.1	4.1	3.1	-8.9	3.3
position A	0.8	1.6	1.4	1.0	-2.5	3.7
position B	-0.2	1.3	1.0	0.8	-2.7	2.3

All values denote the relative dosimetric deviation between motion compensated and the stationary reference measurement in %. Position A refers to chamber positions indicated by solid lines, position B to chamber positions indicated by thin lines in fig. 1c.

### Biological cells samples (RBE-weighted dose)

**Figure 5:**
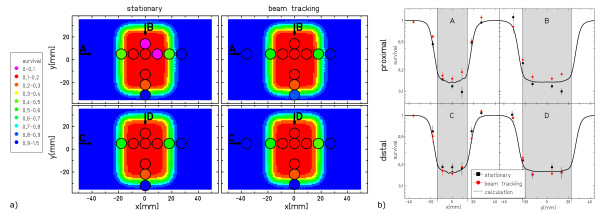
**Results of the biological dosimetry**. Nominal vs. measured survival of the CHO-cell irradiations. (a) The 2D color-wash distribution indicates the survival level predicted by treatment planning. The circles indicate the scored MicroWell plate positions. (b) Along the directions indicated as arrows in (a) profiles are taken showing experimental vs. calculated data (solid line). The experimental data points show the mean survival level of the three measurements, error bars indicate the standard error. The shaded area indicates the target volume.

**Figure 6:**
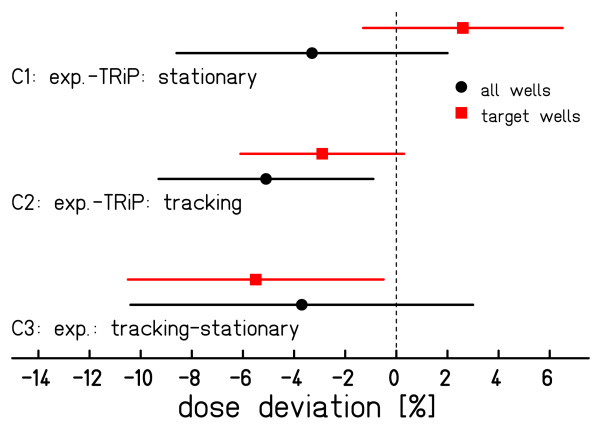
**Result of the RBE-effective dose comparisons**. Each data point is the mean difference in RBE-effective dose. Error bars indicate the standard error. To exclude the influence of well positions in dose gradients, a separate analysis for wells within the target volume was performed.

Figure 5 shows the results of the CHO-cell experiment. Both, stationary measurement as well as beam tracking yield good agreement with the expectation from treatment planning. Largest deviations are seen in wells located in the lateral field gradient (see also profiles in fig. 5b). The data of the comparisons C1-C3 are shown in figure 6. The spread of the results around zero is compatible with the standard errors of the measurements.

## Discussion

Beam tracking is one of the options to treat tumors that are subject to respiratory motion with scanned ion beams. The presented data demonstrate that beam tracking is a feasible and accurate motion mitigation technique.

Small deviations between data from tracking and stationary reference irradiations most likely result from the experimental setup accuracy and the precision of the detector systems. In case of the cell survival experiments, the latter is dominating due to the complex cell processing procedure, including several cell handling steps, and the inherent biological variability. A large deviation in data points is observed in the survival points at +13.5 mm (Fig. 5), but this could be due to the limited statistical power of these experiments (3 independent experiments only). Concerning modeling of biological effects that have to be considered for heavy ion irradiation such as carbon beams the accuracy of the local-effect model for the primary beam and its fragments in the therapy relevant energy range has to be considered also for moving targets. Since our investigation focused on the impact of target motion and validation of the beam tracking system rather than validation of the biological modeling we did not include uncertainties of the model into the comparison between experimental and calculated data.

Additional uncertainties are related to induction and measurement of motion trajectories, discretization of the radiological depth compensation, and potentially the temporal response of the system. Since compensation parameters were determined relative to the voltage level measured by the displacement sensor, a shift of the motion table center (mean voltage level, i.e. compensation = 0) with respect to the isocenter leads to a small shift of the dose distribution. This effect is observable in the profiles of film measurements (fig. 2, distal film, beam tracking vs. stationary) and in the measurements with the pinpoint-ionization chambers (detected shift of 0.6 mm). The magnitude of each shift is comparable to the 0.75 mm shift reported previously [7]. In principle this alignment uncertainty could be further reduced by a more precise motion phantom and improved alignment tools for the heavy water tank (~25 kg). Positioning accuracy of the MicroWell plates is estimated to be less than 1 mm and comprises both the alignment uncertainty of the container and the positioning precision of the plates within the container. Precision of the radiological depth compensation with the energy modulation system is currently limited by digitization to 0.16 mm water-equivalence for communication between therapy control system and controller of the energy modulation system [11]. At least parts of the measured deviation in Bragg-peak depth (~250 *μ*m) can be attributed to this technical limitation. However, in comparison to typical range uncertainties [17] this residual deviation is small; nonetheless it would be possible to decrease the step size by improving the communication if required by future applications. The temporal response of the system was studied in detail by Saito et al. [11]. For lateral compensation the system response is below 1 ms which is much faster than typical irradiation times of 10 ms per spot and thus has a negligible impact on the experimental results. Range compensation is slower. A systematic communication delay of 16 ms plus a mechanical motion delay of for example 11 ms for 5 mm WE range change is required. Since we used motion prediction for the range adaptation component the limited response time of the range modulation device can be mitigated. The results of the depth dose distribution measurements shown in fig. 3 show the feasibility of accurate range adaptation.

Possible systematic uncertainties such as film developer conditions, differences between film batches, entrance position of the range telescope, *W-*value, and positioning of ionization chambers within the water phantom are not relevant because beam tracking performance was compared to stationary reference measurements within the same experimental series. Random uncertainties are present in film analysis (1 mm pixel size in digitization process, 3 mm FWHM beam spot for coordinate system), in the positioning accuracy of the range telescope (10 *μ*m stepping motor step size) [16], and due to accumulated background in the ionization chamber measurements which Karger et al. reported to be 0.5 - 1 mGy/min leading to ~ 0.1% uncertainty in our measurements (2 Gy in ~2.5 min) [15]. In addition, the mechanical precision of the ramp-shaped absorber, the container of the cell samples as well as the wedge-system of the energy modulation system has to be considered which can be estimated to be in the range of 0.1-0.2 mm (each). Biological variability leads to mean standard errors of 4% and 5% for the moving and the stationary setup which is comparable to previous cell survival experiments [14].

For future clinical use of a beam tracking system, larger uncertainties can be expected due to well-known deviations resulting from patient positioning [23] range deviations [17], and motion detection. The impact of these uncertainties on beam tracking will be subject of further research.

In the current status, the beam tracking system is capable to irradiate treatment plans of e.g. liver cancer patients that do not show large range variations. To further advance towards clinical use of ion beam tracking more work is required mainly concerning accurate and precise motion detection, robust treatment planning, and potentially with respect to an improved range modulation system as recently proposed by Chaudhri et al. [24]. It has been reported by several authors that tumor motion characteristics change over the course of treatment [9,25]. Feasible mitigation strategies have to be developed because such changes might alter the dose distribution of dedicated 4D treatment plans applied by beam tracking. In the next step, serial 4DCT patient data will be analyzed and possible techniques to mitigate interfractional changes will be investigated. Besides adequate target dosage, effective doses deposited proximal of the target could be considered. As demonstrated with film experiments, if target motion is compensated by beam tracking inverse interplay effects in proximal regions can lead to over-dosage [4] that should ideally be considered for dose distributions of proximal tissues or even organs-at-risk.

Adequate performance of the motion monitoring system will be as important as the technical precision of the beam tracking system. Several research groups are working on precise motion monitoring and motion prediction techniques; motion detection in the < 2 mm range has recently been reported [26,27]. Sawant et al. achieved a geometrical precision of < 1 mm for a multi-leaf collimator based tracking system that obtains motion information from a Calypso system [26]. Lin et al. used principal component analysis to track lung tumors in fluoroscopic images and reported mean localization errors of less than 1 mm with a maximum of 2.5 mm in 12 patients [27]. For systems that rely on implanted fiducials, like the electro-magnetic Calypso system [28] or fluoroscopy tracking based on radio-opaque markers [8], the compatibility with ion beams has to be evaluated with respect to functionality of the beacon transponders in high-LET fields and considering the dosimetric effect. Considering the data reported for motion detection, it seems feasible to detect, model, and predict target motion in quasi real-time sufficiently accurate to allow tracking with particle beams.

## Conclusions

Ion beam tracking has been fully integrated in the treatment control system at GSI. The system allows target motion detection and simultaneous lateral and radiological depth compensation of target motion in quasi real-time. Validation measurements were performed with radiographic films, a range telescope, an array of ionization chambers, and CHO-cell samples to incorporate the biological effect of carbon ions. Tracking target motion with a scanned particle beam results in dose distributions that are comparable to stationary reference irradiations. To further advance towards clinical use of beam tracking, research has to be performed with respect to motion detection as well as robust 4D treatment planning.

## Competing interests

The *Moving Targets *project at GSI is in part funded by Siemens Healthcare, Particle Therapy. ER and AG are employees of Siemens Healthcare, Particle Therapy. ER has the status *Guest researcher *at GSI. All work was performed during the PhD-work of AG at GSI.

## Authors' contributions

Experimental work: CB, AG, NS, NC, DS; Biological dosimetry: AG; Experimental design: CB, AG, NS, ER; Initial draft of manuscript: CB; consulting & supervision: DS, MD, GK, ER; all authors read and approved the final manuscript.
